# Improving the health and well-being of children of migrant workers

**DOI:** 10.2471/BLT.17.196329

**Published:** 2017-10-31

**Authors:** Catherine Jan, Xiaolin Zhou, Randall S Stafford

**Affiliations:** a Centre for Brain and Cognitive Sciences and School of Psychological and Cognitive Sciences, Peking University, 13th floor, Room 1306, No. 52 Haidian Road, Haidian District, Beijing, 100871, China.; b Beijing Key Laboratory of Behaviour and Mental Health, Peking University, Beijing, China.; c Stanford Prevention Research Center, Stanford University School of Medicine, Stanford, United States of America.

The United Nations Convention on the Rights of the Child emphasizes that the states parties to the convention have the responsibility to ensure that children grow up in a family environment with happiness, love and understanding.^1^ There are almost 1 billion migrants worldwide, with 214 million international migrants and another 740 million internal migrants moving within countries.^2^ Migrants with children may leave their children behind while pursuing economic opportunities. Although there are no available data on the total number of children left behind globally, several reports on international migrants reflect the magnitude of this phenomenon. The Regional Thematic Working Group on International Migration including Human Trafficking estimates that in east and south-east Asia, one child is left behind for each adult working abroad.^3^ Similarly, in the Republic of Moldova, the proportion of children younger than 14 years who are left behind is estimated to have increased from 16% to 31% between 2000 and 2004; in Mexico, more than a third of children experience household disruption due to migration.^4^ The number of children left behind because their parents become internal migrants is even greater, particularly in those areas experiencing rapid urbanization.^5^

Economic progress at the national level and more opportunities to escape poverty at the family level has led to an increase in the number of children separated from their parents. While urban-focused development does have benefits, it can also disrupt family structures and compromise the health and well-being of migrant families. The absence of parents and the challenges of rural life often leave these children particularly vulnerable to major health risks. While grandparents often take on child-rearing tasks, this makeshift solution falls short of optimal parenting.

Parent-child separation has a direct and immediate impact on a child’s physical, cognitive, mental and emotional well-being. Mounting evidence indicates that children separated from their parents fare worse in health outcomes.^6^ Furthermore, adverse childhood events correlate positively with worse educational outcomes, adoption of risky behaviours, development of chronic mental and physical disease in adulthood, and suicide.^7^ Childhood separation has a cross-generational effect that may continue to harm future generations.^8^ This problem exists in high-income countries such as the Netherlands, in middle-income countries such as China and India and in low-income countries.^9^ In response, the United Nations *Transforming our world: the 2030 agenda for sustainable development*^10^ through its sustainable development goals (particularly SDG 17)^11^ recognizes the importance of migration and calls on countries to implement research, policies and practices safeguarding the rights and well-being of migrant workers and their children.

To mitigate the adverse effects of migration on migrant families and pursue the agenda’s ambitious goals, many governments and child protection systems are increasingly adopting a holistic approach that focuses on poverty reduction, family-oriented education programmes, community support, early identification of risks and provision of specialist services for vulnerable children and their families. Strategies that may improve health outcomes include providing antenatal care, parental leave, child allowance for all families, nursery school for all children aged one to six years (as in some European countries), free medical care for all preschool children (as in Japan) and incentives for health-care professionals to practice in rural regions (as in Australia and New Zealand).^9^

China is an extreme case that reveals the challenges that children of internal migrants face. While China’s unprecedented economic growth has vastly improved income in rural areas, urban income has increased disproportionately and rural-urban differences in wages and quality of life have widened. This pattern is associated with the largest internal migration in history, with a total of 274 million rural-to-urban migrants in 2014.^5^ As a result, 61 million rural children (or a quarter of the country’s children) have been left behind by their parents between 2010 and 2014.^5^ This problem is expected to worsen with China’s recent expansion to a two-child policy and continued government investment in urban economic development.

As elsewhere, the structural forces driving rural to urban migration in China include more job opportunities, higher wages, greater access to globalized goods and services, higher quality of housing and the status of being a city-dweller. Migrant workers often leave their children behind due to the lack of affordable child care and education close to the parents’ place of work and because of concerns about safety in cities. Chinese urban migrants include workers across the socioeconomic spectrum, although lower-income migrants face the greatest challenges.

The extent of the problem of left-behind children in countries such as China can be better understood if health is considered “a state of complete physical, mental and social well-being, and not merely the absence of disease or infirmity,” as defined by the World Health Organization. The Global Burden of Disease 2013 study reports that self-harm is the second leading cause of death among 10–24 year-olds, after road injuries.^12^ Suicide rates in rural Chinese youth are threefold that of their urban counterparts,^13^ with these rural-urban differences likely underestimated, given that self-harm in China’s rural children may go unreported and undocumented. Left-behind children in China are at greater risk of insecurity, depression and anxiety,^6^ and are 60% (estimate based on a survey of 840 children) more likely to consider suicide^14^ compared to children living with their parents.

The quality of health-care services for rural children of Chinese urban migrants is also disconcerting. Village doctors and schools are the first point of contact when these children encounter health issues. Lack of adequate training for village doctors and teachers, however, continues to hinder early detection and satisfactory care. For instance, 43% of psychiatrists in China only have three years of technical school training or less.^15^ Furthermore, the cultural stigma associated with mental health conditions represents yet another barrier to rural dwellers seeking care.

Encouragingly, China has initiated key programmes in the past two years aimed at providing better health services to the children left behind. The National Mental Health Work Plan (2015–2020) aims to establish psychological counselling rooms in all schools and to increase awareness of psychological well-being.^16^ In March 2017, the National Health and Family Planning Commission (formerly Ministry of Health) issued a notice emphasizing the health-care needs of the left-behind children and setting guidelines for future funding, surveys and public education.^17^ According to China’s recent thirteenth, five-year plan, setting up a medical system built on a strong foundation of primary care and prevention services is a top government priority. While awaiting the outcome of such new systems, stakeholders should consider a holistic approach to the health of children that includes integrating mental health services into primary health care to improve access to quality care for the youngest and most vulnerable children.

Despite this progress, challenges remain. Strategies to date fail to address the structural causes that create this disadvantaged group of children. Low incomes, poor housing and the marginalized status of migrant workers prevent children from joining their parents. For those who might have sufficient financial means, China’s unique Hukou, the nationwide household registration system, denies most rural children access to urban education and health care. This system was originally designed to regulate rural exodus and to serve as a basis for resource allocation to selected groups of populations. With rapid urbanization over the past 30 years, Hukou does not limit urban migration anymore, but effectively relegates migrants to a second-class status. Urban-dwelling children and adults from rural areas do not receive the same health and educational resources as children from urban areas. This hinders implementing universal health coverage in China. For example, the best performing hospitals in China are currently all state-owned and almost exclusively located in major cities. While people with a rural Hukou may seek care at these hospitals, they cannot access the same preferential subsidies and benefits that are available to local urban residents. This, coupled with the huge urban-rural income and resource discrepancies, leaves very little quality health access for people with a rural Hukou. This internal passport system may thwart approaches shown to be effective elsewhere, including in Australia, Japan and some countries in Europe.^9^ China faces additional barriers, including lack of parental leave for migrant workers, lack of social security for their children and ill-defined land use rights that make it difficult for farmers to sublease land and that necessitate leaving family members behind to watch the land.

Experience from other countries suggests that a multisector approach is required to overcome these barriers; such an approach must be tailored to address the specific needs of migrant families in China. While providing additional health-care services will solve some of the downstream problems, more initiatives must be taken to address the root of this issue: to reduce the growing number of children left behind by their migrant worker parents. We propose substantially increased societal investment in two complementary approaches. First, government needs to promote socioeconomic development in rural areas to help reduce migration to cities in the long term. Second, government needs to create greater opportunities for children to join their parents in urban areas. This may be accomplished by policies aimed at improving the living standards of migrant workers in urban areas and by reducing the discrepancies imposed by the Hukou system, so that children from rural areas gain equal access to urban education and health care. Despite its specific challenges, China benefits from pragmatic policy-making that relies on demonstration cities for policy testing and greater allocable resources controlled by a strong government.

Policies in China and other countries must not only improve the health outcomes in children of migrants, but also reduce the number of left-behind children. Strong initiatives are needed and failure to act decisively will mean that children are not only left behind by their parents, but also by the institutions meant to safeguard their well-being.
